# Pazopanib, a novel multi-kinase inhibitor, shows potent antitumor activity in colon cancer through PUMA-mediated apoptosis

**DOI:** 10.18632/oncotarget.13753

**Published:** 2016-12-01

**Authors:** Lingling Zhang, Huanan Wang, Wei Li, Juchang Zhong, Rongcheng Yu, Xinfeng Huang, Honghui Wang, Zhikai Tan, Jiangang Wang, Yingjie Zhang

**Affiliations:** ^1^ College of Biology, Hunan University, Changsha, China; ^2^ Department of Laboratory Medicine, Xiangya School of Medicine, Central South University, Changsha, China; ^3^ Department of Internal Medicine, The Third Xiangya Hospital, Central South University, Changsha, China; ^4^ School of Life Sciences, The Chinese University of Hong Kong, Shatin, New Territories, Hong Kong, China; ^5^ Department of Veterinary Medicine, College of Animal Sciences, Zhejiang University, Hangzhou, China; ^6^ Department of Radiology, The Third Xiangya Hospital, Central South University, Changsha, China; ^7^ Shenzhen Institute, Hunan University, Shenzhen, China

**Keywords:** colon cancer, PUMA, apoptosis, Akt, p53

## Abstract

Colon cancer is still the third most common cancer which has a high mortality but low five-year survival rate. Novel tyrosine kinase inhibitors (TKI) such as pazopanib become effective antineoplastic agents that show promising clinical activity in a variety of carcinoma, including colon cancer. However, the precise underlying mechanism against tumor is unclear. Here, we demonstrated that pazopanib promoted colon cancer cell apoptosis through inducing PUMA expression. Pazopanib induced p53-independent PUMA activation by inhibiting PI3K/Akt signaling pathway, thereby activating Foxo3a, which subsequently bound to the promoter of PUMA to activate its transcription. After induction, PUMA activated Bax and triggered the intrinsic mitochondrial apoptosis pathway. Furthermore, administration of pazopanib highly suppressed tumor growth in a xenograft model. PUMA deletion in cells and tumors led to resistance of pazopanib, indicating PUMA-mediated pro-apoptotic and anti-tumor effects *in vitro* and *in vivo*. Combing pazopanib with some conventional or novel drugs, produced heightened and synergistic antitumor effects that were associated with potentiated PUMA induction via different pathways. Taken together, these results establish a critical role of PUMA in mediating the anticancer effects of pazopanib in colon cancer cells and provide the rationale for clinical evaluation.

## INTRODUCTION

Colon cancer makes major contribution to cancer mortality in the world [1, 2]. Although significant improvement of therapeutic approach and the application of targeted therapy in past decades, median overall survival of patients with metastatic colon cancer is 2 years after chemotherapy [3, 4]. The five year survival for colorectal cancer patients is very low, which is less than 10%, mainly because of the drug resistance during therapy. Colon cancer develops from the acquisition of genetic mutations, which leads the action of signals to eliminate apoptosis and confer resistance to drugs [5–10]. In this regard, most clinical drugs for cancer therapy are chosen by their ability to induce apoptosis.

Pazopanib is an orally bioavailable tyrosine kinase inhibitor (TKI) which selectively targets against multi-receptors [11–14]. Pazopanib was a FDA approved drug for kidney cancer therapy and has previously shown clinical activity against several other cancer types [15–22], including colon cancer [23–25]. Pazopanib has significantly prolonged progression-free survival (PFS) benefit in patients with advanced or metastatic carcinoma [21, 26, 27], which is widely established as first-line therapy with limited side-effects. It has also been used as a second-line therapy and showed clinical activity as well as tolerated after failure of other systemic treatments in advanced neuroendocrine tumor (NET) [28] as well as in non-small cell lung cancer (NSCLC) [29]. Preclinical evaluation showed pazopanib has excellent efficacy of antiangiogenic and antitumor activity [14] in relapsed or refractory colorectal cancer [23]. However, the mechanisms by which pazopanib exercise its antitumor activity especially how to induce cell apoptosis are poorly understood.

PUMA (p53-upregulated modulator of apoptosis), a pro-apoptotic factor, belongs to the Bcl-2 family and functions as an inducer of apoptosis in several cancer cells [30]. PUMA is lowly expressed in normal cells and tissues, which could be upregulated by DNA-damaging stimulation or p53 activation [31–33]. After induction by apoptotic stimuli, PUMA triggers Bax/Bak mitochondrial membrane translocation and activates these pro-apoptotic signals by neutralizing anti-apoptotic proteins of Bcl-2 family [31, 34–36], leading mitochondrial outer membrane permeabilization (MOMP), caspase cascade and cell apotpsosis. PUMA promoted apoptosis in p53-dependent and -independent manner after many kinds of stimulation [37]. PUMA was initially identified as a p53 target gene that is induced upon DNA-damaging agents through induction of p53 [31]. Later, PUMA was found to be up-regulated by non-DNA-damaging agents via other transcription factors like FoxO3a (Forkhead Box O3a), which is independent of p53 [38–40], p73 [41, 42], and NF-kB [43, 44]. For its function, PUMA seems to facilitate tumor suppression, because PUMA knockdown activates oncogenic factors and accelerates cancer development [45]. Knocking out PUMA in human colon cancer cells [30, 31, 46–49], or in mice abrogated mitochondrial apoptosis induced by various stimuli. In contrast, over-expressing PUMA accelerates apoptotic process in various types of cancer cells [31, 32, 50, 51].

In the present study, the antitumor effects of pazopanib were analyzed in colon cancer *in vitro* and *in vivo* (xenograft mice). The results show that pazopanib suppressed colon cancer growth directly by inducing PUMA expression. The expression level of PUMA was enhanced through FoxO3a, after the inhibition of PI3K/Akt signal. PUMA deletion resulted in resistance to pazopanib-induced apoptosis both in colon cancer cells and in xenografts. Taken together, these results suggest PUMA induction as an indicator of the therapeutic efficacy. They also provide an anticancer mechanism of pazopanib, and imply one of the potential strategies contributing to chemotherapeutic resistance in tumors.

## RESULTS

### Pazopanib induced p53-independent PUMA expression in colon cancer cells

**Figure 1:**
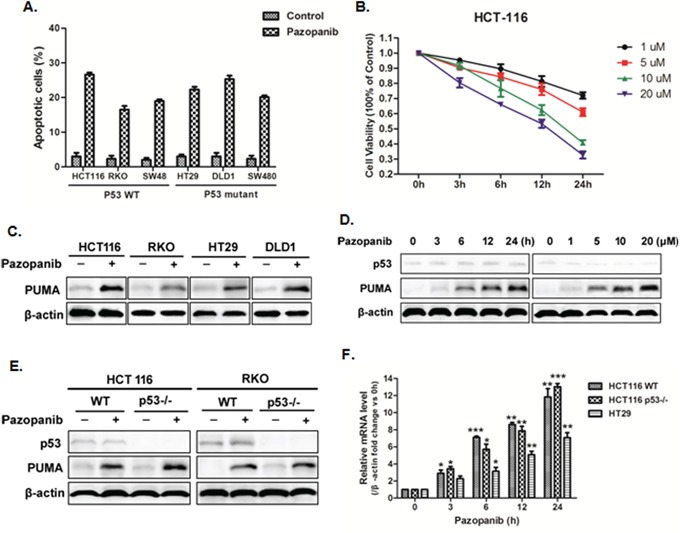
Pazopanib promoted cell apoptosis and PUMA induction in colon cancer cells **(A)** Cell apoptosis was analyzed in various colon cancer cells after 20 μM pazopanib treatment for 24 hours by counting condensed and fragmented nuclei after incubation with Hoechst 33342. The percentage of apoptotic cells were used to calculate. **(B)** Cell viability was analyzed using Cell Counting Kit-8 at 0, 3, 6, 12 and 24 hours after 1, 5, 10, or 20 μM pazopanib treatment in HCT-116 cells. Data represent the mean ± SEM of four independent experiments. **(C-F)** Pazopanib increased p53-independent PUMA expression in various colon cell lines. (C) Western blotting analysis of PUMA expression in indicated colon cancer cell lines treated with 20 μM pazopanib for 24 hours. (D and E) The expression of p53 or PUMA by western blotting analysis in (D) HCT-116 cells treated with (left) 20 μM pazopanib for indicated hours or (right) with 1-20 μM pazopanib for 24 hours or in (E) WT, p53^-/-^ HCT-116 or RKO cells treated with 20 μM pazopanib for 24 hours. (F) PUMA mRNA induction by pazopanib was analyzed in WT, p53^-/-^ HCT-116 or HT-29 cells by real-time qPCR and normalized to the housekeeping gene β-actin. The values are the mean ± SEM (n=3) from a representative experiment. *P<0.05, **P<0.01, **P<0.001 vs. 0h.

We first test whether pazopanib can induce apoptosis or not in colon cancer cells. As shown in Figure 1A, pazopanib caused significant cell apoptosis in all analyzed colon cancer cells, including WT and p53 mutant cells. To establish a proper dose of pazopanib in our system, cell viability was detected in HCT-116 cells at indicated time points after 1-20 μM pazopanib treatments. The result showed cell viability decreased over time and showed negative correlation with drug dose (Figure 1B), suggesting pazopanib inhibited cell proliferation in a time and dose dependent way.

To explore whether PUMA plays an important role in the response to pazopanib, we first detect PUMA expression in WT (HCT-116, RKO) and p53 mutant (HT-29, DLD1) colon cancer cell lines. As shown in Figure 1C, pazopanib markedly induced PUMA expression in all of these cell lines, which was time and dose dependent (Figure 1D). PUMA induction was also observed in both WT and p53^-/-^ HCT-116 cells (Figure 1E), suggesting p53-independent PUMA expression by pazopanib. Of note, p53 expression had no change through the whole process (Figure 1D). The mRNA level of PUMA was also enhanced in colon cancer cells with different p53 statuses (Figure 1F), which is prior to PUMA protein accumulation. Taken together, these data indicated that pazopanib increased PUMA expression by transcriptional activation in a p53-independent manner.

### FoxO3a transcriptionally activated PUMA following Akt inhibition by pazopanib

**Figure 2:**
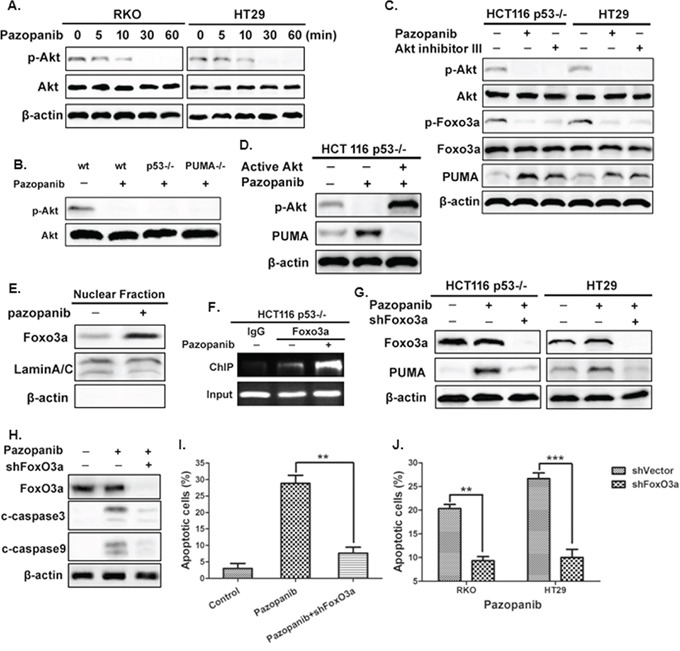
The Akt/FoxO3a axis regulated PUMA induction by pazopanib **(A)** and **(B)** Akt is inhibited after pazopanib treatment in colon cells. The expression of P-Akt (S473) was detected by western blotting in (A) RKO or HT-29 cells after 20 μM pazopanib treatment for indicated times or in (B) WT, p53^-/-^ or PUMA^-/-^ cells in the presence/absence of 20 μM pazopanib. **(C)** and **(D)** Akt suppression is associated with PUMA induction by pazopanib. (C) The expression of p-Akt (S473), p-FoxO3a (S253) and PUMA were detected after the treatment of 20 μM pazopanib in p53^-/-^ HCT-116 and HT-29 cells in the presence/absence of Akt inhibitor III. (D) HCT-116 p53^-/-^ cells were transfected with either empty vector or a constitutively-active Akt expression constructs for 24 hours, following 24 hours of 20 μM pazopanib treatment. The levels of p-Akt (S473) and PUMA were analyzed by Western blotting. **(E-G)** FoxO3a bound directly with PUMA and transcriptionally activated of PUMA expression in response to pazopanib. (E) FoxO3a translocated to nucleus after pazopanib treatment. Levels of FoxO3a expression after pazopanib stimulation in the nuclear fractions were detected by western blotting. LaminA/C and β-actin were used as the nucleus and cytoplasm marker for loading, respectively. (F) Chromatin immunoprecipitation (ChIP) was performed on p53^-/-^ HCT-116 cells after 12 hours pazopanib treatment. IgG was used to as a control for the FoxO3a-specific antibody. (G) FoxO3a and PUMA were detected in p53^-/-^ HCT-116 or HT-29 cells following the treatment of pazopanib, with FoxO3a knockdown or not. Similar results were obtained from three independent experiments. **(H-J)** The effects of Foxo3a knockdown on cell apoptosis. Cells were treated with pazopanib for 24 hours, with FoxO3a knockdown or not. (H) The expression of cleaved caspase3 and caspase9 were analyzed by western blotting. (I and J) Cell apoptosis was detected by nuclear staining with Hoechst 33342 in (I) HCT-116 or in (J) RKO and HT29 cells. **P<0.01, ***P<0.001.

PI3K/Akt, a common pathway downstream of multiple kinases, triggers cancer initiation and development. We first investigated whether Akt could be suppressed by pazopanib. As shown in Figure 2A, the phosphorylation level of Akt decreased in both RKO and HT-29 cells after different time points of pazopanib treatment. De-phosphorylation of Akt also occurred in p53^-/-^ and PUMA^-/-^ cells after pazopanib stimulation (Figure 2B), indicating Akt inactivation by pazopanib is independent of p53 and PUMA. Furthermore, blockage of Akt signal by pazopanib or by Akt inhibitor increased PUMA expression in regardless of p53 status (Figure 2C). While over-expression of active Akt decreased PUMA expression, even in the presence of pazopanib, in p53^-/-^ cells (Figure 2D). These strongly suggested pazopanib induced PUMA expression probably through inhibition of Akt signal in colon cancer cells.

In addition, we found FoxO3a, a well-established Akt substrate, was activated by dephosphorlation after pazopanib treatment (Figure 2C). Nuclear translocation was also occurred in response to pazopanib (Figure 2E), indicating FoxO3a may serve as a transcriptional factor to activate PUMA. ChIP analysis showed increased recruitment of FoxO3a to the region of PUMA promoter after pazopanib stimulation (Figure 2F). Furthermore, knockdown of FoxO3a by shRNA highly suppressed PUMA activation (Figure 2G) and cell apoptosis (Figure 2H–2J and Figure S1B) induced by pazopanib. These indicated that pazopanib-mediated PUMA induction and cell apoptosis are dependent on Akt/FoxO3a signaling pathway.

### PUMA is indispensable in pazopanib-induced apoptosis

**Figure 3:**
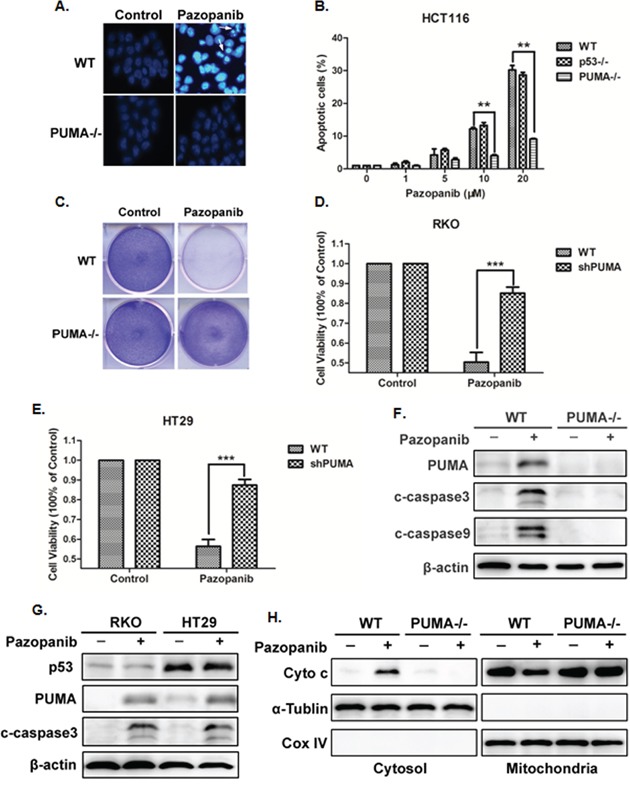
Pazopanib induced PUMA-dependent apoptosis **(A)** and **(B)** Hoechst 33342 morphological examination of apoptosis in WT, p53^-/-^ or PUMA^-/-^ HCT-116 cells. Cells were treated with 20 μM pazopanib and incubated for 24 hours, then stained with Hochest 333342. (A) Representative pictures of the cells. (B) Relative apoptosis was calculated by counting condensed and fragmented nuclei normalized by untreated cells. Similar results were obtained from three independent experiments. Data were obtained from 3 independent experiments. *P<0.05, **P<0.01 vs. WT. **(C)** Colony formation of WT and PUMA^-/-^ HCT-116 cells by crystal violet staining after 24 hours of pazopanib treatment. **(D)** and **(E)** Cell viability was analyzed by CCK-8 after PUMA knockdown or not in (D) RKO cells or in (E) HT-29 cells after pazopanib treatment for 24 hours. **(F-H)** Pazopanib induced mitochondrial pathway apoptosis in colon cells. (F and G) Western blotting showing the expression of p53, PUMA and cleaved caspase3 or caspase9 in (F) WT and PUMA^-/-^ HCT-116 cells or in (G) RKO and HT-29 cells after 20 μM pazopanib treatment. (H) The release of mitochondrial cytochrome c was analyzed by Western blotting. The cytoplasm and mitochondria fraction were isolated from WT or PUMA^-/-^ HCT-116 cells treated with 20 μM pazopanib for 24 hours. α-tublin and CoxIV were used as the cytosolic and mitochondrial fraction marker for loading, respectively. Similar results were obtained from three independent experiments.

To examine whether PUMA triggers pazopanib-induced apoptosis, cell apoptosis was detected in HCT-116 WT and PUMA^-/-^ cells in response to paozopanib. As shown in Figure 3A and 3B, WT cells, but not PUMA^-/-^ cells, showed obvious cell apoptosis and chromatin condensation after paopanib stimulation. Consistent with this observation, PUMA^-/-^ cells had highly improved survival than WT HCT-116 cells in response to pazopanib in a long-term colony formation assay (Figure 3C). Furthermore, PUMA knockdown (shPUMA) in RKO and HT-29 cells also showed significantly increased cell viability compared with that of WT cells in response to pazopanib (Figure 3D and 3E).

Activation of caspase-3 was found with the induction of PUMA expression in WT HCT-116 cells (Figure 3F), which was also occurred in both WT (RKO) and mutant p53 (HT-29) cells (Figure 3G), suggesting the probable mitochondrial-dependent but p53-independent apoptosis by pazopanib. As expected, PUMA deficiency abolished pazopanib-induced cytochrome c release (Figure 3H) and caspase-3 activation (Figure 3F). These findings indicated that PUMA was indispensible in pazopanib-triggered colon cancer cells apoptosis.

### Pazopanib induced apoptosis via PUMA/Bax axis

**Figure 4:**
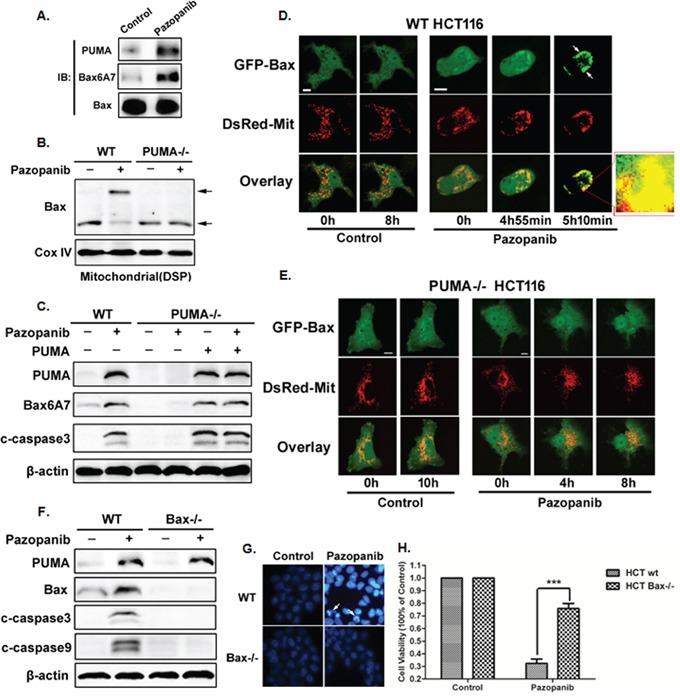
Bax activation was required for pazopanib treatment induced apoptosis **(A-C)** Bax activation and mitochondial translocation is accomplished by binding with PUMA. (A) The interaction between PUMA and Bax was detected by Co-IP. Co-immunoprecipitation with an anti-Bax antibody was used to pull down total Bax, western blotting for PUMA shows the amount of PUMA binding to Bax. Bax conformational change was detected with anti-Bax 6A7 (activated) antibody. (B) Bax multimerization in mitochondria fraction was analyzed by Western blotting after DSP cross-link. (C) Transfecting PUMA into PUMA^-/-^ HCT-116 cells or not, the expression of PUMA, activated Bax and cleaved caspase3 were determined by western blotting. **(D)** and **(E)** Bax translocation induced by pazopanib in WT but not in PUMA^-/-^ HCT-116 cells. Real-time detection the dynamic GFP-Bax translocation in (D) WT or (E) PUMA^-/-^ HCT-116 cells co-transfected with GFP-Bax and DsRed-Mit with/without pazopanib treatment. The image in the mark frame was the zoom-in image of the frame indicated region. **(F-H)** The essential role of Bax in pazopanib-induced apoptosis. (F) PUMA, cleaved caspase3 or caspase9 were detected in WT or Bax^-/-^ HCT-116 cells after pazopanib for 24 hours. (G and H) Hoechst 33342 morphological examination of apoptosis in WT or Bax^-/-^ HCT-116 cells. (G) Representative pictures of the cells. (H) Relative apoptosis was calculated by counting condensed and fragmented nuclei normalized by untreated cells. Similar results were obtained from three independent experiments. ***P<0.001 vs. WT.

Our previous report showed PUMA induced apoptosis via activating Bax both directly and indirectly [52]. Herein, we analyzed Bax activity and its relationship with PUMA in HCT-116 cells. As shown in Figure 4A, PUMA interacted with activated Bax (6A7) after pazopanib treatment. In addition, Bcl-xL had enhanced binding with PUMA, but decreased binding with Bax following pazopanib stimulation (Figure S2A), suggesting the indirect activation of Bax by PUMA through competitive binding to Bcl-xL. Thus over-expression of Bcl-xL reduced pazopanib-induced apoptosis, while knocking down Bcl-xL induced much more apoptosis (Figure S2B).

Next, Bax oligomerization and activation were detected in HCT-116 WT and PUMA^-/-^ cells. The results showed that pazopanib effectively induced Bax oligomerization (Figure 4B) and activation (Figure 4C) in mitochondrial only in WT HCT-116 cells but not in PUMA^-/-^ cells. Importantly, over-expression PUMA could restore Bax activation and apoptosis induced by pazopanib in PUMA^-/-^ cells (Figure 4C), suggesting the essential role of PUMA for Bax activation. This is further confirmed by real-time monitoring mitochondrial translocation of GFP-Bax in living cells (Figure 4D and 4E) and endogenous Bax translocation by western blotting (Figure S2C).

Finally, to explore the function of Bax in pazopanib-triggered apoptosis, Bax^-/-^ cells were recruited. As shown in Figure 4F, PUMA expression was increased in both WT and Bax^-/-^ cells after pazopanib treatment, whereas the expression of cleaved-caspase3/9 and chromatin condensation (Figure 4G) were suppressed in Bax^-/-^ cells compared to that in WT cells. These indicated that Bax was an essential inducer for cell apoptosis and growth inhibition by pazopanib, which was further confirmed by the CCK8 analysis (Figure 4H).

### PUMA mediated therapeutic responses to pazopanib

**Figure 5:**
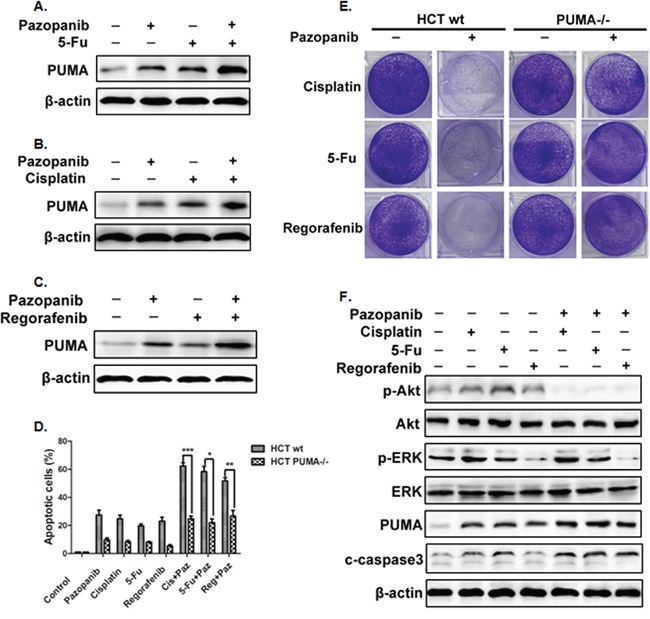
PUMA increased the chemosensitization effects of pazopanib by inducing apoptosis **(A-C)** Western blotting analysis of PUMA expression after the treatment of 10 μM pazopanib in combination with (A) 20 mg/mL 5-FU or (B) 40 uM Cisplatin or (C) 20 mM Regorafenib alone, or their combinations for 24 hours in HCT-116 cells. **(D)** Apoptosis was analyzed by nuclear staining with Hoechst 33342 in WT or PUMA^-/-^ HCT-116 cells treated with pazopanib, 5-FU, Cisplation, or Regorafenib alone or their combinations as in A. Data represent the mean ± SEM of four independent experiments. *P<0.05, **P<0.01, ***P<0.001 vs. WT. **(E)** Colony formation of HCT-116 cells treated with different drugs alone or combined with pazopanib. WT and PUMA^-/-^ HCT-116 cells were treated with 40 μM cisplatin, 20 mg/mL 5-FU or 20 mM regorafenib alone or in combination of 10 μM pazopanib for 24 hours, followed with crystal violet staining of attached cells at 14 days. **(F)** The expression of P-Akt, P-ERK (Thr202/Tyr204), PUMA, cleaved-caspase3 were detected by western blotting after the treatment of 40 μM cisplatin, 20 mg/mL 5-FU or 20 mM regorafenib alone or in combination of 10 μM pazopanib for 24 hours in HCT-116 cells. Similar results were obtained from three independent experiments.

Pazopanib is commonly used combination with other antitumor drugs. Previous results showed PUMA-dependent apoptosis induction in response to pazopanib (Figure 3). Together with the pro-apoptotic role of PUMA in colon cancer cells, we would like to know whether combined with other drugs could synergize to promote apotosis by pazopanib. Here, we combined pazopanib with three drugs that could induce PUMA expression and apoptosis, like DNA-damaging drug 5-FU and cisplatin or the TKI inhibitor regrafenib. We found that combination of pazopanib with these drugs induced much higher level of PUMA expression (Figure 5A–5C). Besides, combination treatment induced much more apoptosis than the sum of single treatment (Figure 5D). The analogous results were obtained in long term colony assay (Figure 5E). In contrast, apoptosis induced by combined treatment was greatly suppressed in PUMA^-/-^ cells compared with that in WT cells (Figure 5D), suggesting PUMA is required for combination treatment-triggered apoptosis. Importantly, 5-FU and cisplatin induced obvious increase of phosphor-Akt, whereas regorafenib greatly reduced the phosphorylation level of ERK (Figure 5F). When compared with single treatment, the combination treatment led to obvious decrease of Akt activity but no obvious change of phosphor-ERK. Together, these results indicated PUMA is indispensible during pazopanib chemosensitization by Akt inhibition, and robust induction of PUMA is indicative of effective drug combinations.

### The antitumor effects of pazopanib *in vivo* are PUMA dependent

**Figure 6:**
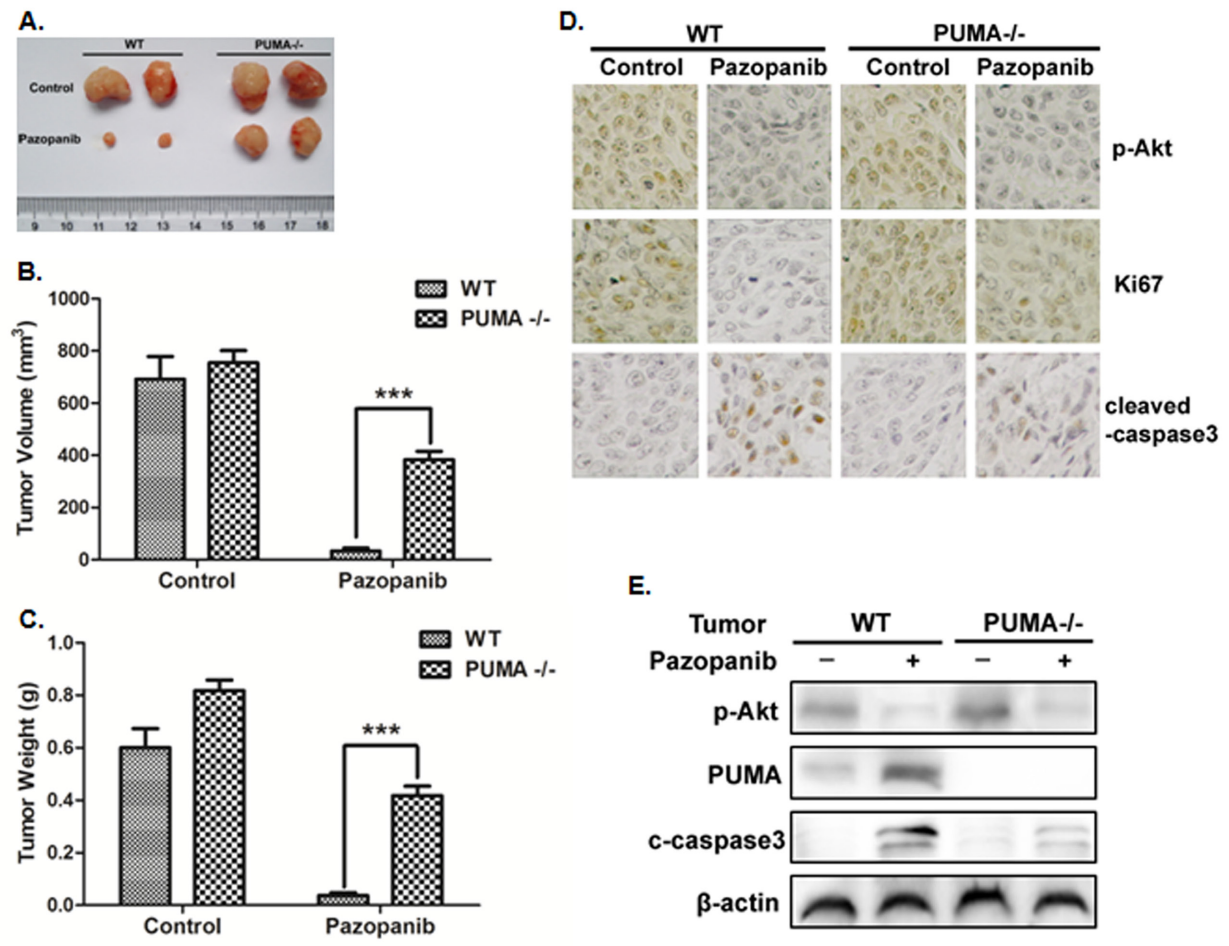
PUMA exhibited the antitumor effects of pazopanib *in vivo* Nude mice were injected s.c. with 1 × 10^6^ WT or PUMA^-/-^ HCT-116 cells. Once the tumor was measurable, mice were treated daily with 30 mg/kg pazopanib by oral gavage, for 15 consecutive days. **(A)** Representative tumors at the end of the experiment. **(B)** Tumor volume and **(C)** tumor weight at indicated time points after treatment was calculated (n=6 per group). Statistical significance is indicated for the comparison of pazopanib-treated WT and PUMA^-/-^ tumors. ***P<0.001. **(D)** IHC staining analyzed of P-Akt (S473), ki67 and active Caspase-3 from paraffin-embedded sections of control or treated tumor tissues. **(E)** p-Akt, PUMA, and cleaved-caspase3 expression were analyzed by Western blotting in representative tumors. Similar results were obtained from three independent experiments.

Previous study showed that pazopanib has tolerability without obvious side effects [53]. To clarify if PUMA mediates the antitumor effects of pazopanib, we generated subcutaneous tumors using both WT and PUMA^-/-^ HCT-116 cells in a xenograft mice model. Pazopanib caused significant growth suppression in WT tumors (Figure 6A–6C). However, PUMA^-/-^ tumors were less sensitive to pazopanib treatment, which showed modest growth inhibition compared to WT tumors (Figure 6A–6C). In WT tumors, P-Akt and ki67 expression reduced, while PUMA and cleaved-caspase3 expression increased after pazopanib treatment (Figure 6D and 6E). However, the reduction of ki67 expression and activation of caspase3 were not seen in PUMA^-/-^ tumors, although Akt activity was also inhibited (Figure 6D and 6E), indicating PUMA-dependent antitumor effects of pazopanib.

## DISCUSSION

Pazopanib, a potent and selective multi-targeted receptor TKI, has been clinically used in a variety of tumor types [17–20]. However, little is known about how pazopanib inhibits growth of cancer cells. Here, we report efficacy and mechanistic studies of pazopanib in colon cancer. We found that pazopanib showed antitumor activity and suppressed colon cancer growth both *in vitro* and *in vivo*. Using multiple cell lines, we show for the first time that tumor suppression by pazopanib is dependent on PUMA induction. Specially, pazopanib showed highly inhibition of Akt activity and induced PUMA through transcription factor FoxO3a, but not p53. This p53-independent PUMA expression activated Bax and the mitochondrial apoptotic pathway.

Using multi colon cancer cells including WT, p53 mutant or knockout cells, we found pazopanib could induce all of these cells apoptosis with no difference, during which PUMA expression was highly increased (Figure 1), indicating the potential role of PUMA during this apoptosis. Targeting some activated anti-apoptotic kinase is a strategy for the development of anti-tumor drugs. The most commonly changed kinases are ERK and Akt signal in response to Tyrosine kinase inhibitors (TKIs). Our results showed Akt activity is greatly suppressed in wide type, p53^-/-^ or PUMA^-/-^ colon cancer cells following pazopanib treatment (Figure 2A and 2B), suggesting Akt signal is independent of or in the upstream of p53 and PUMA. Furthermore, inhibition of Akt by pazopanib or by inhibitor significantly increased PUMA expression (Figure 2C), while over-expression of active Akt suppressed this PUMA induction (Figure 2D). In contrast, the changes of phosphor-ERK by paozopanib were not uniform, which reduced in RKO and HT-29 cells, but did not change in HCT-116 and DLD1 cells (Figure S3 and S4). Interestingly, suppression of ERK by its inhibitor in RKO and HT29 cells did not lead to PUMA induction (Figure S1B), strongly suggesting that ERK inhibition may not contribute to PUMA induction. However, regorafenib did decrease p-ERK and increase PUMA expression in HCT-116 cells, which is consistent with other group's results [54]. Thus it seems the relation between ERK and PUMA is complicated which needs to be further studied.

Previous study showed direct induction of PUMA by NF-kB after aurora kinase inhibition in colon cancer cells [55]. The unchanged of phosphor-p65 after pazopanib treatment (Figure S4) suggested that NF-kB signaling is not involved in our experiment model. Besides, some other studies reported that p73 is involved in PUMA activation [41, 42]. However, our results showed that p73 expression did not change during PUMA induction by pazopanib, suggesting p73-independent PUMA expression in our experiment model. Notably, FoxO3a was activated and then translocated to nucleus, where it bound to the promoter of PUMA to stimulate its transcription (Figure 2F). Knocking down FoxO3a by shRNA greatly reduced PUMA expression (Figure 2G), caspase-3/9 activation and cell apoptosis by pazopanib (Figure 2H–2J and S1B). Together, these indicate that PUMA induction is through FoxO3a activation upon Akt inhibition during pazoapnib-induced apoptosis, which is p53-independent.

Till now, reliable biomarker of pazopanib's efficacy in colon cancer is lacking. Our results demonstrated that pazopanib induced PUMA expression in all analyzed colon cells regardless of p53 status (Figure 1C–1F). This PUMA induction is indispensible for pazopanib-induced apoptosis. Firstly, we found that pazopanib induced cyto c release, Bax activation, caspase3 and 9 cleaved and cell apoptosis. However, all these apoptotic events did not occur in PUMA^-/-^ or PUMA knockdown cells (Figure 3 and 4). These indicated that pazopanib induced PUMA-dependent mitochondrial pathway apoptosis. Further experiments demonstrated PUMA promoted Bax activation by binding to Bax directly and by binding to Bcl-xL competitively after pazopanib stimulation (Figure 4A and S2A). Finally, *in vivo* xengraft experiments further indicated PUMA-triggered cell apoptosis is essential for the antitumor effects of pazopanib (Figure 6). Therefore, PUMA up-regulation might become a potential biomarker for response of colon cancer to pazopanib.

Bim induction and Mcl-l degradation were observed in colon cancer cells in response to sunitinib, another TKI. Our data showed that Bim is induced only in RKO cells but not other analyzed colon cancer cells (Figure S4 and S5). For Mcl-1, its expression significantly decreased in HCT-116, HT-29 and DLD1 cells but only had slight reduce in RKO cells (Figure S4 and S5A). Knockdown experiments showed that Bim was not involved in Bax activation and apoptosis induced by pazopanib (Figure S5B and S5C). Further study is required to determine whether other Bcl-2 family members are correlated with cell apoptosis induced by pazopanib.

One big problem for the application of chemotherapeutic agents in clinic is the acquired resistance [56, 57]. Thus, it is important to identify effective combination therapies to prevent drug resistance. In this study, we used the combination of pazopanib with two conventional drugs 5-FU and cisplatin or with another novel TKI regorafenib. 5-FU induced p53-dependent PUMA expression [34, 58]. We found that Akt activation contribute to 5-FU resistance in our previous study [59]. Thus inhibition Akt activity by pazopanib in combined treatment synergistic the effect of 5-FU on cell apoptosis (Figure 5A and 5F). Cisplatin also induced p53-dependent PUMA expression in SKOV3 cells [60] or renal tubular cells (RTC) [61, 62]. Together with our results that pazopanib increased p53-independent PUMA expression in colon cancer cells (Figure 1), we could explain the synergistic effects of cisplatin on pazopanib-induced apoptosis maybe due to the synergy of PUMA up-regulation through p53-dependent and -independent ways. Regorafenib, a small molecule inhibitor for the Ras/Raf/MEK/ERK pathway, has been reported to increase PUMA expression in colorectal cancer cells. As shown in Figure 5C–5F, the combined treatment with pazopanib and regorafenib did suppress ERK and Akt activities, and led to much more PUMA induction. However, as we discussed before, further investigation is required to confirm the relation between ERK and PUMA.

In conclusion, our results demonstrated that pazopanib exhibited direct anticancer activity towalirds colon cancer cells. This antitumor effect of pazopanib is PUMA-dependent, progressing from Akt inhibition, FoxO3a activation and nuclear translocation, leading to PUMA induction and onset of mitochondrial apoptosis. Together with the data from xenograft mice *in vivo*, we provide novel mechanistic insights for anti-tumor mechanism of pazopanib and have important implications for further investigation in future clinical trials.

## MATERIALS AND METHODS

### Cell culture and treatments

The human colon cancer cell lines HCT-116, RKO, HT-29, DLD1, SW48 and SW480 were obtained from American type culture collection (ATCC). HCT-116 with p53, PUMA, or Bax null cell lines were kindly provided by Dr Bert Vogelstein (Johns Hopkins University, Baltimore, MD, USA). All the cell lines were cultured in McCoy's 5A modified media, supplemented with 10% fetal bovine serum (FBS), penicillin (100 units/ml), and streptomycin (100 mg/ml) in 5% CO_2_ at 37°C in humidified incubator. For treatment, anticancer agents or inhibitors chemicals were added in the medium directly before detection. Transfections were performed with Lipofectamine™ 2000 reagent according to the manufacturer's protocol. The medium was replaced with fresh culture medium after 5 hours. Cells were then examined at 24–48 hours after transfection.

### Antibodies and reagents

Primary antibodies against p53, Phospho-Akt (S473), total-Akt, Phospho-FoxO3a (S253), total-FoxO3a, Phospho-ERK (Thr202/Tyr204), total-ERK, PUMA, Bax and cleaved Caspase3, β-actin were purchased from cell signaling; α-tubulin antibody was from Santa Cruz Technologies. Lipofectamine™ Reagent was purchased from Invitrogen. HRP-conjugated anti-rabbit and or anti-mouse secondary antibodies an ECL-plus kit were from GE Healthcare. 5-FU was purchased from APP Pharmaceuticals. Cisplatin and regofenib were purchased from Axon Medchem. Other chemicals were mainly from Sigma. The plasmid of expressing PUMA was kindly supplied by Jian Yu, Ph.D. [31]. The oligonucleotide for shFOXO3a was synthesized as 5′-CACCGACTCCGGGTCCAGCTCCACTTCAAGA GAGTGGAGCTGGACCCGGAGTTTT TTTG-3′.

### Cell viability and apoptosis assays

Colon cells were cultured in 96-well microplate at a density of 5 × 10^3^ cells/well for 24 hours. Cell viability was assessed with CCK-8 (Dojindo Laboratories Kumamoto, Japan) at indicated time post-treatment according to the manufacturer's instructions. OD450, the absorbance value at 450 nm, was read with a 96-well plate reader (DG5032, Hua dong, Nanjing, China), to determine the viability of the cells.

For analysis of apoptosis by nuclear staining, cell apoptosis was morphologically evaluated with Hoechst 33342. For analysis of apoptosis by nuclear staining, colon cells were cultured on the coverslip of a chamber, rinsed with phosphate-buffered saline (PBS) and then 500 ml DMEM containing 5 μg Hoechst 33342 was added in, incubated at 37°C with 5% CO_2_ for 15 minutes. Apoptosis was assessed through microscopic visualization of condensed chromatin and micronucleation.

For colony formation assays, equal number of cells after different treatments were plated into 6-well plates. Colonies were visualized by crystal violet staining 14 days after plat.

### Western blotting and subcellular fraction

Western blotting analysis was performed as previously described [63, 64]. To detect Cytochrome c release, cytoplasmic and mitochondrial fractions were isolated by Mitochondrial Fractionation Kit (Active Motif) by differential centrifugation according to the manufacturer's instructions. Both cytoplasmic and mitochondrial fractions of Cytochrome c were then detected by Western blotting.

To detect Bax multimerization, purified mitochondrial fractions were cross-linked with DSP (dithiobis(succinimidyl propionate)) (1 mmol/L), followed by Western blotting analysis.

To detect Bax mitochondrial translocation, cytosolic and mitochondrial fraction were isolated from cells treated with pazopanib using Cell Compartment Kit (Qproteome, Germany) according to manufacturer's instructions with minor modifications, and probed by Western blotting for Bax.

To detect Foxo3a nuclear translocation, HCT-116 cells was firstly treated with pazopanib for 3 hours. Nuclear fraction was isolated from cells using Cell Compartment Kit (Qproteome, Germany), and probed by Western blotting for Foxo3a.

### Co-immunoprecipitation

To detect the interaction between PUMA and Bax, about 4 ml of Bax antibodies were firstly added to 400 ml cell lysates. The mixtures were mixed on a rocker at ambient temperature for 2 hours. The immunocomplexes were captured by the addition of protein G/A-agarose (Roche Applied Sciences, Indianpolis, IN 46250-0414) mixed at 1:10 ratio, followed by incubation at ambient temperature for 1 hour. The beads were washed three times by PBS and then collected by centrifugation at 12,000 rpm for 5 seconds. After the final wash, the beads were mixed with 60 ml of 2× Laemmli sample buffer, heated at 100°C for 5 minutes, and analyzed by Western blotting using PUMA, Bax6A7 or Bax antibody.

### Chromatin immunoprecipitation

ChIP assay was performed using the Chromatin Immunoprecipitation Assay kit (Millipore, Massachusetts, USA) according to manufacturer's instructions with minor modifications. All the solutions used were from this ChIP Assay kit unless otherwise stated.

Briefly, after pazopanib treatment, P53^-/-^ HCT-116 cells were fixed with 1% formaldehyde and lysed in warm SDS lysis buffer. The genomic DNA was obtained and sheared to 200–1,000 bp by sonicationonice. Samples were pre-cleared with Protein A Agarose/Salmon Sperm DNA (50% Slurry) for 1 hour at 4°C with agitation. Then anti-Foxo3a antibody was added and incubated overnight on a shaker at 4°C. Normal rabbit IgG (Invitrogen) was used as a negative control. The protein A agarose/salmon sperm DNA (50% slurry) bead was then added to precipitate the antibody/protein/DNA complexes. After washed with serial wash buffers, DNA-protein immunocomplexes were eluted from the beads by elution buffer (1% SDS, 0.1 M NaHCO3) for 30 minutes. Finally, the protein-DNA cross-links were reversed to release DNA by incubation with 0.2 M NaCl at 65°C for 4 hours.

The total DNA was finally recovered from the samples by phenol/chloroform extraction and ethanol precipitation. Semi-quantitative PCR was then performed as describe above. Primer sequences used in our experiments were listed as follows: Proximal primers: 5′-CGCGCCTCTCCAAACCCCGC-3′; Distal primers: 5′-CTCCGTGCCGCCCCCCCGCC-3′.

### GFP-Bax translocation assay in living cells

To monitor GFP-Bax translocation in living cells, HCT-116 cells were co-transfected with pGFP-Bax and pDsRed-Mit. Using Zeiss LSM 510 confocal microscope, we imaged both the distribution pattern of GFP-Bax and that of DsRed-Mit simultaneously after pazopanib treatments as previously described [64]. Bax redistribution was assessed by the matching fluorescence of GFP-Bax and DsRed-Mit emission. The cells exhibiting strong punctate staining of GFP, which overlapped with the distribution of DsRed, were counted as the cells with mitochondrially localized Bax.

For time-lapse imaging, culture dishes were mounted onto the microscope stage that was equipped with a temperature controlled chamber (Zeiss). During control experiments, bleaching of the probe was negligible.

### Real-time reverse transcription-PCR

Total RNA was extracted with Tri-Reagent (Molecular Research Center, Cincinnati, OH) according to the manufacturer's protocol and our previous report [65]. In brief, the amount and purity of the RNA were determined by spectrophotometry, and 3 μg of RNA from the colon cancers after pazopanib treatment were used in each RT reaction. Real-time qPCR was performed as we previously described on C1000 Thermal Cycler CFX96 Real-time PCR Detection System (Bio-Rad) according to our previous report [65].

### Xenograft mouse model and treatment

WT and PUMA^-/-^ HCT-116 xenografts were established and measured as described [66]. Female 5- to 6-week-old nude mice (Vital River, China) were housed in a sterile environment with micro isolator cages and allowed access to water and chow ad libitum. 1 × 10^6^ cells were resuspended in 100 μl of PBS (phosphate-buffered saline solution) and injected subcutaneously into the flanks of nude mice. Once the tumor was measurable, mice were given daily 30 mg/kg pazopanib or vehicle control by oral gavage. Pazopanib was resuspended in 0.5% hydroxypropyl methylcellulose (HPMC) (Sigma-Aldrich) and 0.1% Tween-80 (Sigma-Aldrich) in water as a vehicle (pH 1.3-1.5) before use. Mice were treated for 7 days a week, and terminated after 15 days treatment. Tumor growth was monitored by calipers, and tumor volumes were calculated according to the formula 0.5 × length × width^2^. Mice were euthanized when tumors reached ~1.0 cm^3^ in size.

### Autopsy and histopathology

Animals were autopsied when the tumor reached to the maximal size and tissues were collected and examined. Experimental and control tissue samples were fixed in 10% neutral-buffered formalin 24 hours, and washed once with 1X PBS and then transferred into 70% ethanol and stored at 4°C. Tissues were proceed by ethanol dehydration and embedded in paraffin by Lecia according to standard protocols. Sections (5 μm) were prepared for immunohistochemisty according to our previous study [59]. In breif, after antigen retrieval with citric acid (pH 6.0) endogenous peroxidase activity was blocked with 1% hydrogen peroxide. Primary antibody (anti-P-Akt (Ser473), Ki67 and c-caspase3 antibody, dilution 1:200) was applied and incubated with secondary anti-bodies conjugated to peroxidase labeled dextran polymer. Sections not exposed to secondary antibody served as negative controls.

### Statistical analysis

Statistical analyses were carried out using GraphPad Prism V software. All assays were repeated independently for a minimum of three times. Data are represented as mean ± SEM in the figures. P values were calculated using the Student's paried t-test. Differences were considered statistically significant at *P<0.05, **P<0.01, ***P<0.001.

## SUPPLEMENTARY MATERIALS FIGURES



## Abbreviations

PUMA: p53 up-regulated modulator of apoptosis
Bax: Bcl-2-associated X protein
CCK-8: Cell Counting Kit-8
CRC: colorectal cancer
