# Supplementary material for: Pazopanib, a novel multi-kinase inhibitor, shows potent antitumor activity in colon cancer through PUMA-mediated apoptosis
Source: Oncotarget. 2016 Dec 1;8(2):3289–303. doi: 10.18632/oncotarget.13753 (PMC5356882; doi:10.18632/oncotarget.13753)
Supplement: Supplementary file 1 [file oncotarget-08-3289-s001.pdf]

Pazopanib, a novel multi-kinase inhibitor, shows potent antitumor activity in colon cancer through PUMA-mediated apoptosis

SUPPLEMENTARY FIGURES

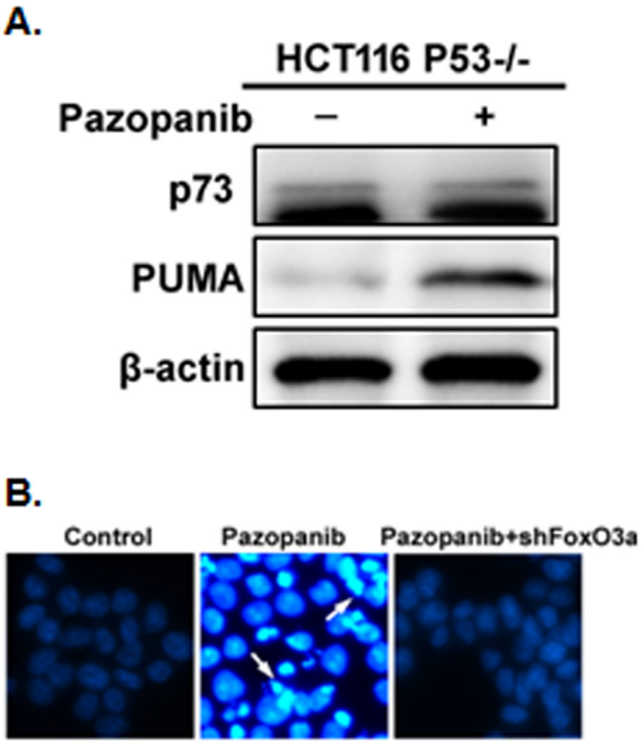

Supplementary Figure S1: Fox03a but not p73 is involved in PUMA induction by pazopanib in p53-/- HCT-116 cells. **A.** Western blot showing the expression of p73 and PUMA with pazopanib treatment or not. **B.** Hoechst 33342 morphological examination of apoptosis in HCT-116 cells transfected with shFoxO3a or not.

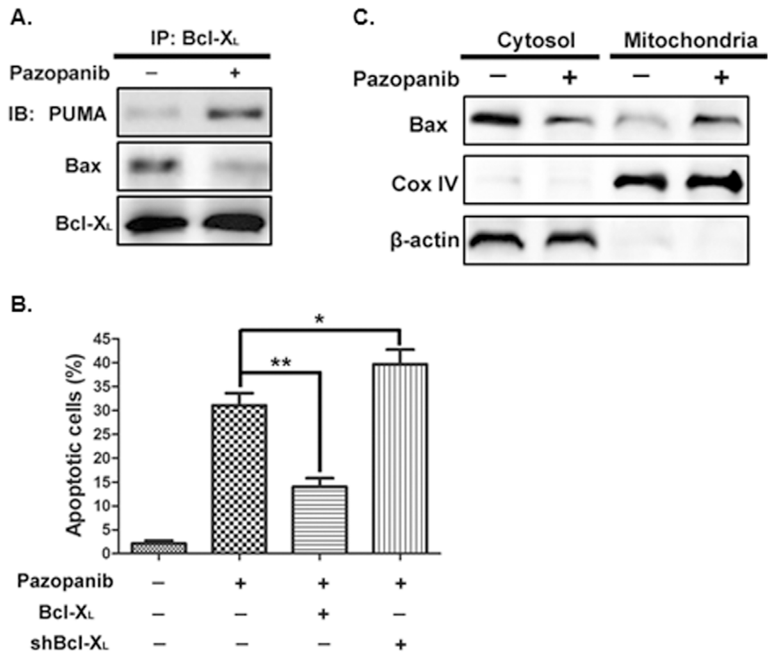

Supplementary Figure S2: PUMA indirect activate of Bax by competitive binding to Bcl-xL. A. The interaction between Bcl-xL and Bax, as well as between PUMA and Bcl-xL were detected by Co-IP. Co-immunoprecipitation with an anti-Bcl-xL antibody was used to pull down total Bcl-xL, western blotting for PUMA and Bax shows the amount of PUMA or Bax binding to Bcl-xL. **B.** The effects of over-expressing or knocking down Bcl-xL on cell apoptosis in HCT-116 cells treated with pazopanib. Relative apoptosis was calculated by counting condensed and fragmented nuclei normalized by untreated cells. Similar results were obtained from three independent experiments. Data were obtained from 3 independent experiments. *P<0.05, **P<0.01 vs. Control. **C.** Bax translocation was analyzed by Western blotting in mitochondria and cytosol fractions. β-actin and CoxIV were used as the cytosolic and mitochondrial fraction marker for loading, respectively. Similar results were obtained from three independent experiments.

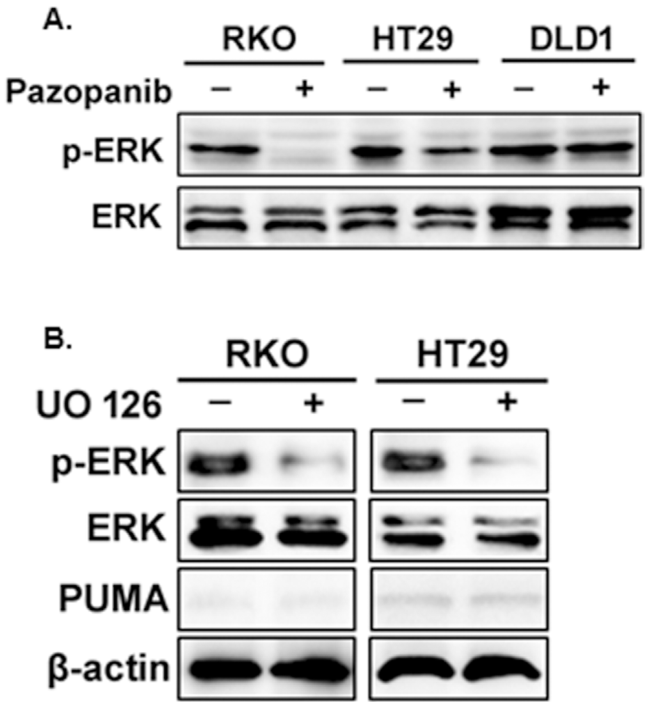

Supplementary Figure S3: ERK inhibition is not attributable to PUMA induction in colon cancer cells. A. The expression of p-ERK was detected by western blotting in RKO, HT-29 or DLD1 cells after 20 μM pazopanib treatment. **B.** The expression of p-ERK and PUMA in RKO and HT-29 cells in the presence/absence of UO126. Similar results were obtained from three independent experiments.

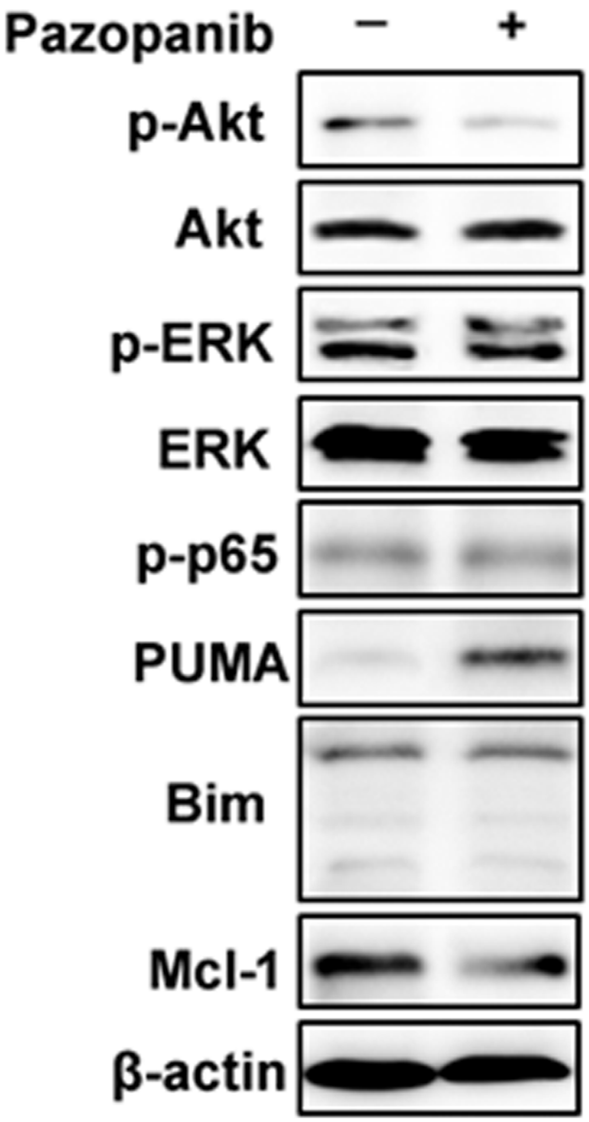

Supplementary Figure S4: p65 and Bim are not required in PUMA induction and apoptosis in HCT-116 cells. The levels of p-Akt, p-ERK, p-p65, PUMA, Bim and Mcl-1 were analyzed by western blotting. Similar results were obtained from three independent experiments.

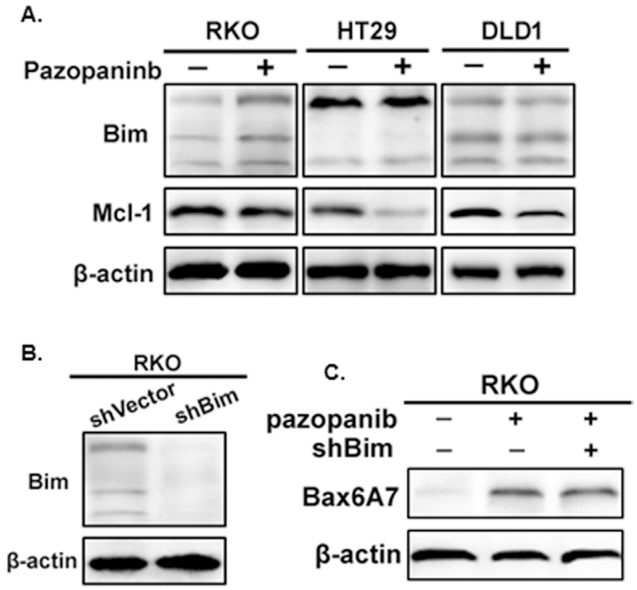

Supplementary Figure S5: Bim is not involved in pazopanib-induced apoptosis in colon cancer cells. A. The levels of Bim and Mcl-1 were detected by western blotting in RKO, HT-29 and DLD1 cells in the presence/absence of pazopanib. **B.** and **C.** Bax activation was detected following pazopanib treatment, with Bim deletion or not. (B) Confirmatory experiment of Bim knock-down in RKO cells. (C) Western blotting showing activated Bax (Bax6A7) in RKO cells transfected with shBim or not. Similar results were obtained from three independent experiments.
